# I-shaped incisions for papilla reconstruction in second stage implant surgery

**DOI:** 10.5051/jpis.2010.40.3.139

**Published:** 2010-06-25

**Authors:** Eun-Kwon Lee, Yeek Herr, Young-Hyuk Kwon, Seung-Il Shin, Dong-Yeol Lee, Jong-Hyuk Chung

**Affiliations:** 1 Department of Periodontology, Kyung Hee University School of Dentistry, Seoul, Korea.; 2 Institute of Oral Biology, Kyung Hee University School of Dentistry, Seoul, Korea.

**Keywords:** Dental esthetics, Dental implants, Dental papilla

## Abstract

**Purpose:**

Pink gingival esthetic especially on the anterior teeth has been an important success criterion in implant-supported restoration. Inter-implant papillae are a critical factor for implant esthetics, and various techniques for inter-implant papilla reconstruction have been introduced. The aim of this study is to suggest and evaluate a surgical technique for reconstructing inter-implant papillae.

**Methods:**

A 28-year-old man had an implant placed on the #13 and #14 area. Four months after implant placement, a second stage surgery was planned for inter-implant papilla reconstruction. At the time of the abutment connection, I-type incisions were performed on the #13i & #14i area followed by full-thickness flap elevation and connection of a healing abutment on underlying fixtures without suture.

**Results:**

Two weeks after the second stage implant surgery, soft tissue augmentation between the two implants was achieved.

**Conclusions:**

I-shaped incisions for papilla reconstruction performed during the second stage implant surgery were useful for inter-implant papilla reconstruction and showed a good esthetic result.

## INTRODUCTION

Dental implants are now considered a routine treatment modality for replacing missing teeth in the majority of dental applications [1]. However, to reconstruct a natural soft tissue appearance between two implants in the anterior part of the maxilla is complex and challenging [2,3]. Nowadays, pink gingival esthetic has become a hot issue for most clinicians and has been a critical factor in deciding the overall success of the implant-supported restoration [4,5]. The soft tissue profile is one of the most important factors of the esthetic implant-supported restoration; thus clinicians should consider esthetic problems caused by loss of inter-implant papillae in anterior regions. The absence of the inter-implant papilla can lead to cosmetic deformities, phonetic difficulty, and food impaction [6-8]. However, reconstructing a predictable peri-implant papilla is the most complex and challenging aspect of implant dentistry. In particular, when two or more adjacent implants are placed, surgical techniques to reconstruct inter-implant papillae show predictably low results [9], and loss of the vertical dimension of the edentulous ridge may further complicate papilla reconstruction. Although many attempts have been made to reconstruct inter-implant papillae with various surgical techniques, the reconstruction of the papilla adjacent to the dental implant is still difficult to perform and often unpredictable [4,10-12].

Various techniques for reconstructing inter-implant papillae are suggested at the time of second-stage surgery. Palacci [3] suggested the method of rotating the pedicle flap to the mesial side of the healing abutment followed by a semilunar beveled incision. Grossberg [9] modified Palacci's method with a horizontal incision creating a double-pedicle flap. Nemcovksy et al. [4] performed a U-shaped incision with divergent arms open toward the buccal side of the implant site, which creates a double-pedicle flap. Each part of the buccal flap was sutured over de-epithelized papillae. Azzi et al. [13] reconstructed the interproximal papillae by undermining the papillae from their insertion to the bone followed by insertion of connective tissue into the pouch-like tunnel. Misch et al. [14] proposed a 'split-finger' surgical method in which three interlacing finger-like incisions were made and each of the fingers was sutured over the desired inter-implant papilla position. Tinti and Benfenati [15] suggested a ramp mattress suture, which pulls the buccal flap coronally, to obtain a papilla between two implants in the buccal area. Shahidi et al. [16] introduced a new flap design and a sutureless technique for papilla reconstruction, and reported good esthetic results by performing a U-shaped incision open in the mesial direc tion. After incision, the flaps were elevated minimally and healing abutments were connected to plump up the soft tissue that formerly covered the implant.

This case modified the method introduced by Shahidi et al. [16] Four months after the implant placement on the maxillary right canine and first premolar, we performed second stage implant surgery with the purpose of reconstructing the inter-implant papilla.

## CASE DESCRIPTION

**Figure 1:**
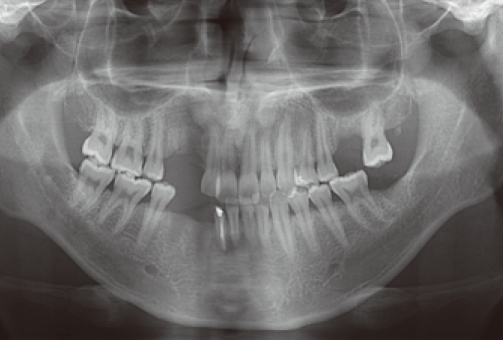
Preoperative panoramic view. Residual root on #13 is observed.

**Figure 2:**
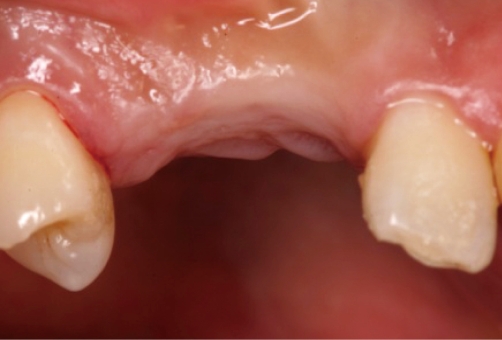
Four months after implant placement: facial view.

**Figure 3:**
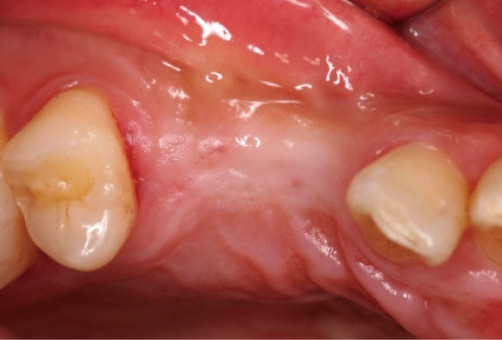
Four months after implant placement: occlusal view.

**Figure 4:**
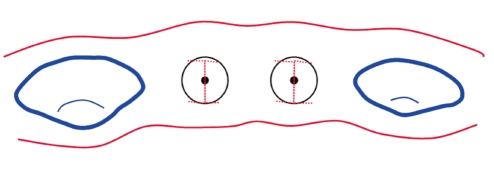
Schematic drawing of I-type incision. Labial horizontal incision: 0.5-1 mm inside from the border of implant. Vertical incision: middle line. Palatal horizontal incision: border of implant.

**Figure 5:**
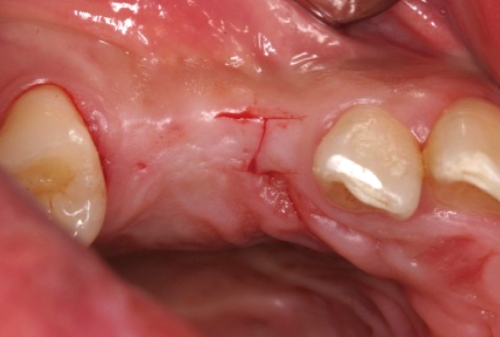
I-incision on #13i.

**Figure 6:**
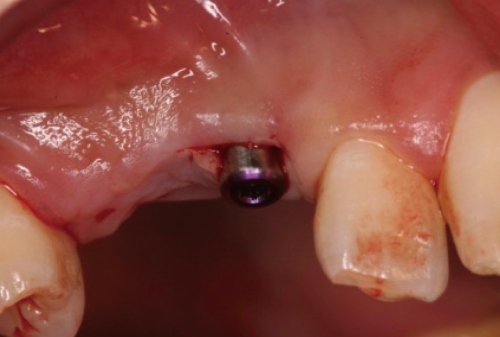
Healing abutment connection on #13i.

**Figure 7:**
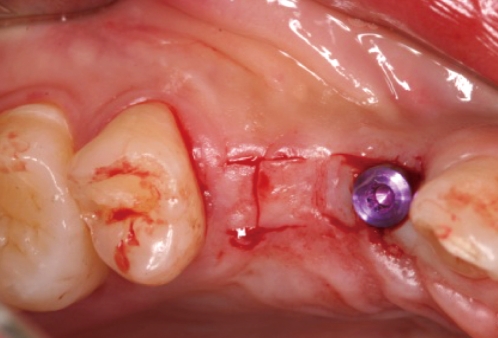
I-incision on #14i.

**Figure 8:**
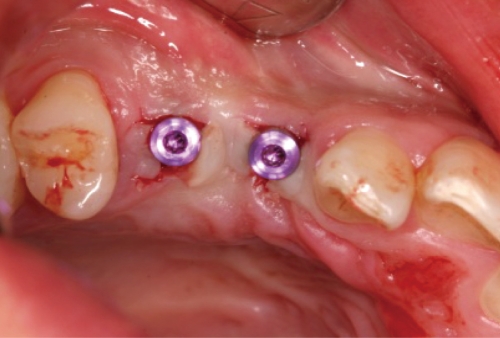
Healing abutment connection on #14i: occlusal view.

**Figure 9:**
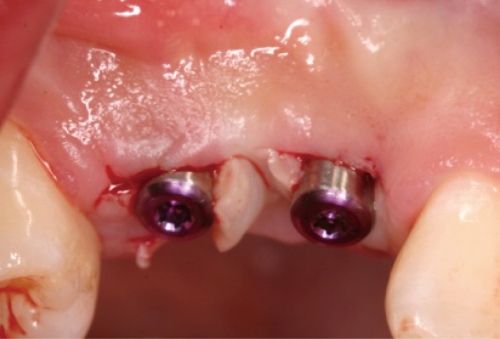
Healing abutment connection on #14i: facial view.

**Figure 10:**
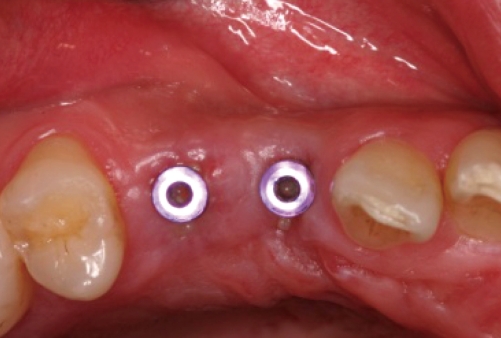
Two weeks after second-stage surgery: occlusal view.

**Figure 11:**
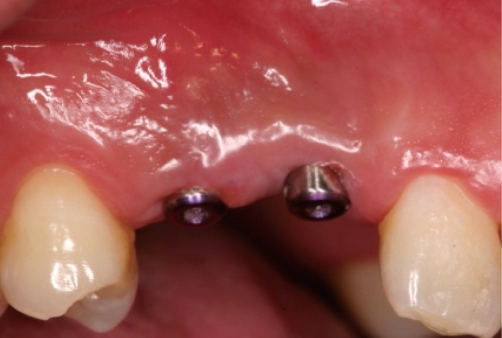
Two weeks after second-stage surgery: facial view.

A 28-year-old male who was in good systemic health visited the Department of Periodontology, School of Dentistry, Kyung Hee University, to have implants placed in the edentulous area of #13, #14, #26, #37, #43, #44. Number 13 was in the root rest state and immediate implant placement surgery was planned (Fig. 1). The residual root was removed and dental implants (NobelReplace™, Nobel Biocare, Göteborg, Sweden), of which diameter and length were 3.5 mm and 13 mm, were placed in the area of #13 and #14. followed by full thickness flap reflection under local anesthesia. Deprotenized bovine bone (Bio-Oss™, Geistlich Pharma AG, Switzerland), covered by resorbable collagen membrane, (Bio-Gide™, Geistlich Pharma AG) was grafted to the palatal side of the fixtures at the time of surgery because the implant threads were exposed on the palatal side of the fixtures. After a healing period of four months, a second stage implant surgery was planned (Figs. 2 and 3). For the purpose of reconstructing the inter-implant papilla between #13i and 14i, this second stage implant surgery was planned using the method of performing an I-shaped incision, which is a modification of the method suggested by Shahidi et al. [16]. A Labial horizontal incision with a #15 blade was performed mesiodistally 0.5-1.0 mm inside from the labial border of the implant. A horizontal incision was also performed, parallel to the buccal side, on the palatal side, which was in contact with the palatal border line of the implant different from the labial side. Another incision was done bucco-lingually over the implant midline perpendicular to the horizontal incision lines performed on the labial and palatal sides. As a consequence, the final incision line became I-shaped (Figs. 4 and 5). The flap was reflected with care and the implant was exposed to remove the cover screw. The healing abutment has been connected and both flaps were folded up alongside the healing abutment intending them to heal without suture (Fig. 6). The same incision was also performed on #14i (Fig. 7) followed by a healing abutment connection (Figs. 8 and 9). Antibiotics and analgesics were each administered for five days, and a mouth rinse with 0.12% chlorhexidine was recommended for the following two weeks. Two weeks after surgery, the healing was uneventful and soft tissue augmentation between the two implants was seen (Figs. 10 and 11).

## DISCUSSION

Gingival esthetics has become an important success criterion for implant-supported restoration. Unesthetic implant restoration, therefore, is considered to be a failure. Especially for the anterior maxilla, esthetic results are quite an important factor for successful restoration and establishment of intact papilla between implant and tooth, or between adjacent implants. The level of inter-implant papilla is influenced by the previous bone level, soft tissue quantity and quality, peri-implant biotypes, implant position, and inter-implant distance [6]. Therefore soft and hard tissue quality and quantity, peri-implant biotype [17], implant diameter, position, and emergence profile [18] should be considered with adequate treatment planning and evaluation of the surgical site prior to implant placement. If needed, ridge augmentation procedures using guided bone regeneration or/and connective tissue grafts are carried out prior to implant placement to attain a more acceptable esthetic result in the inter-implant papillary area. However, the predictable regeneration of the inter-implant papilla remains a complex challenge because most groups of supracrestal fibers do not exist in the gingival tissue surrounding the implant abutment and the blood supply of inter-implant papilla is restricted [19] due to the absence of the periodontal ligament and the associated blood vessel branches. Four potential time points can be differentiated for soft and/or hard tissue management: prior to implant placement; at time of placement or during the healing phase of the implant; at second-stage surgery; and in the maintenance phase [20]. Various surgical techniques have been suggested to reconstruct inter-implant papilla at the time of second stage implant surgery, but comparison of efficacy among techniques or long-term results is still insufficient, and the procedure is not predictable.

In this case, we tried to reconstruct inter-implant papilla with I-shaped incisions and the sutureless technique, which is a modification of the method suggested by Shahidi et al. [16]. According to the method of Shahidi et al. [16], a U-shaped flap, from the occlusal view, was created by two mesiodistal horizontal incisions and another buccolingual incision perpendicular to them. If multiple implants were placed, the U-shaped incisions were added to the distal side of the most distal implant to form an H-shaped design. The mesiodistal horizontal incision line ended halfway between the implant platform and the adjacent implant or tooth. The buccal horizontal incision formed a parabola buccally at the buccal border of the implant platform to create a gingival margin around the implant.

In this case, we suggest a new method including an I-shaped incision which was done over every implant for our case. To minimize the possibility of labial gingival tissue recession, labial horizontal incision lines were positioned 0.5-1.0 mm inside from the labial border of implants. Also, the horizontal incision is limited to the mesiodistal distance of the implant neck. The flaps were minimally elevated and healing abutments were connected. Each flap was supported by the healing abutments and able to plump up stably.

The advantages of this new method, compared to old ones, are decreased chair time, less postoperative discomfort and improved esthetics. This sutureless method with minimal incision does not decrease blood flow to the overlying flap and it minimized the probability of trauma or inflammatory reaction [21]. Therefore, the above-described surgical technique would be the least invasive one. Two weeks after the second stage implant surgery, the surgical site showed uneventful healing and the patient reported less postoperative discomfort. Comparing to before the surgery, remarkable soft tissue augmentation between the two implants was achieved.

## References

[1] Albrektsson T, Dahl E, Enbom L, Engevall S, Engquist B, Eriksson AR (1988). Osseointegrated oral implants: a Swedish multicenter study of 8139 consecutively inserted Nobelpharma implants. J Periodontol.

[2] Grunder U (1997). The inlay-graft technique to create papillae between implants. J Esthet Dent.

[3] Palacci P, Palacci P, Ericsson I, Engstrand P, Rangert B (1995). Papilla regeneration technique. Optimal implant positioning & soft tissue management for the Branemark system.

[4] Nemcovsky CE, Moses O, Artzi Z (2000). Interproximal papillae reconstruction in maxillary implants. J Periodontol.

[5] Tarnow DP, Eskow RN (1995). Considerations for single-unit esthetic implant restorations. Compend Contin Educ Dent.

[6] Gastaldo JF, Cury PR, Sendyk WR (2004). Effect of the vertical and horizontal distances between adjacent implants and between a tooth and an implant on the incidence of interproximal papilla. J Periodontol.

[7] Tarnow DP, Magner AW, Fletcher P (1992). The effect of the distance from the contact point to the crest of bone on the presence or absence of the interproximal dental papilla. J Periodontol.

[8] Tarnow DP, Cho SC, Wallace SS (2000). The effect of inter-implant distance on the height of inter-implant bone crest. J Periodontol.

[9] Grossberg DE (2001). Interimplant papilla reconstruction: assessment of soft tissue changes and results of 12 consecutive cases. J Periodontol.

[10] Jemt T (1997). Regeneration of gingival papillae after single-implant treatment. Int J Periodontics Restorative Dent.

[11] Beagle JR (1992). Surgical reconstruction of the interdental papilla: case report. Int J Periodontics Restorative Dent.

[12] Kan JY, Rungcharassaeng K (2001). Site development for anterior single implant esthetics: the dentulous site. Compend Contin Educ Dent.

[13] Azzi R, Etienne D, Takei H, Fenech P (2002). Surgical thickening of the existing gingiva and reconstruction of interdental papillae around implant-supported restorations. Int J Periodontics Restorative Dent.

[14] Misch CE, Al-Shammari KF, Wang HL (2004). Creation of inter-implant papillae through a split-finger technique. Implant Dent.

[15] Tinti C, Benfenati SP (2002). The ramp mattress suture: a new suturing technique combined with a surgical procedure to obtain papillae between implants in the buccal area. Int J Periodontics Restorative Dent.

[16] Shahidi P, Jacobson Z, Dibart S, Pourati J, Nunn ME, Barouch K (2008). Efficacy of a new papilla generation technique in implant dentistry: a preliminary study. Int J Oral Maxillofac Implants.

[17] Kan JY, Rungcharassaeng K, Umezu K, Kois JC (2003). Dimensions of peri-implant mucosa: an evaluation of maxillary anterior single implants in humans. J Periodontol.

[18] Pradeep AR, Karthikeyan BV (2006). Peri-implant papilla reconstruction: realities and limitations. J Periodontol.

[19] Berglundh T, Lindhe J, Jonsson K, Ericsson I (1994). The topography of the vascular systems in the periodontal and peri-implant tissues in the dog. J Clin Periodontol.

[20] Hurzeler MB, Weng D (1996). Periimplant tissue management: optimal timing for an aesthetic result. Pract Periodontics Aesthet Dent.

[21] Flanagan D (2002). An incision design to promote a gingival base for the creation of interdental implant papillae. J Oral Implantol.

